# Editorial: Post-pandemic digital realities of older adults

**DOI:** 10.3389/fpsyg.2023.1275257

**Published:** 2023-08-28

**Authors:** Hannah R. Marston, Loredana Ivan, Dennis Rosenberg, Barbara Ratzenboeck

**Affiliations:** ^1^School of Health, Wellbeing and Social Care, The Open University, Milton Keynes, United Kingdom; ^2^Department of Communication, The National University of Political Studies and Public Administration, Bucharest, Romania; ^3^Israel Gerontological Data Center, The Paul Baerwald School of Social Work and Social Welfare, Hebrew University of Jerusalem, Jerusalem, Israel; ^4^Department of Human Services, University of Haifa, Haifa, Israel; ^5^Center of Excellence in Research on Ageing and Care, University of Jyväskylä, Jyväskylä, Finland; ^6^Center for Inter-American Studies, University of Graz, Graz, Austria

**Keywords:** digital realities, older adults, digital use during pandemics, older adults and digital use, digital spectrum and COVID-19

The COVID-19 (SARS-CoV-2) pandemic is to date the phenomenal event of the 21st century and of 2020, leading to global lockdowns and various directives rolled out by governments to protect their citizens (Marston and del Carmen Miranda Duro, 2020; Marston et al., 2023a). A whole new way of life started to unfold for all of us, in which what we had known in the preceding years was no longer the same, and our routines of commuting to work and socializing with friends and family ceased, leisure activities were curtailed, education delivery was transferred onto digital platforms, and many of us were only allowed out of our homes for essential reasons, including shopping (e.g., groceries and collection of medication) or for work purposes (Mandache and Ivan, 2022).

Since the beginning of the 21st century, digital technologies, practices, and transformation have been moving quickly, but the events of 2020 exacerbated this process, leading to services and platforms becoming the primary “go-to” place for everyone who owned a digital device and who had access to the Internet and the digital skills to engage with the platform(s).

This phenomenal event has led scholars from across many disciplines to come together, form new networks, and collaborate on innovative projects (Ivan and Cutler, 2021a; Earle et al., 2022; Ratzenboeck et al., 2022; Marston et al., 2023b; Taipale et al., 2023) in an attempt to capture the lived experiences during 2020 and 2021. The scholarly activity that has been conducted will provide insights for future scholarly historians, social scientists, technologists, and many others who will be curious to understand how digital transformation came into the fore and how people adapted to a new way of living in a post-pandemic society, as well as to learn about the experiences during this time.

The primary focus of this Research Topic is to present and contribute to the discourse of digital technologies and practices during the COVID-19 pandemic.

The articles in this Research Topic are broad, and its topics include (1) robotics (Berridge et al.; Maalouly et al.) from the perspective of the US and Japan; (2) the perceptions of people from the UK on the role of digital companions in reducing loneliness (Martin et al.); (3) Chatbots (Iancu and Iancu); (4) the role of videoconferencing in nursing homes in France (Racin et al.); (5) mobile and wearable technologies (Fowe and Boot); (6) interacting with QR codes and purchasing items using contactless payment options (Morrison et al.); (7) digital exclusion, digital skills (Wilson-Menzfeld et al.) from a UK perspective, digital literacy (Finkelstein et al.) in the context of the US, and digital inclusion (Reuter et al.) observed in Sweden.

These articles add to a growing body of research focusing on this once-in-a-lifetime event of the 21st century (Renu, 2021; Ummer et al., 2021; Vargo et al., 2021; Freeman et al., 2022; Smith et al., 2022), including the wider societal debates of digital technologies and practices by people across the life course and understanding transgenerational perspectives of and interactions with technologies (Marston et al., 2020a, 2022; Ivan and Nimrod, 2021).

Berridge et al. present a study exploring the interest in and use of “companion robots” or “artificial robots” as a way of mitigating loneliness and understanding the ethical issues associated to them. This study recruited 496 people, who ranged in age from 25 to 88 years, and the statistical analysis explored the relationships between age, health, and perceptions toward the impact of loneliness and comfort surrounding deception. The findings showed that 68.7% of participants thought that artificial robots would make them feel less lonely, although nearly 70% reported that the use of artificial robots would make them feel somewhat to very uncomfortable with the idea of making the individual believe that a robot is human. Overall, this study notes how there was no strong belief that artificial robots would alleviate loneliness, and in respect to deception, it is posited that future solutions need to consider design implications to prevent this likelihood.

Maalouly et al. present an experimental study in which older adults tele-operated a robot to get involved in prosocial activities in two experimental situations: engaging in a conversation in which they would give information to visitors about their city and talking with children from a children's center to offer their support. The two situations were used to understand how older adults experienced remote-controlled work, how it was to start a new job in a remote situation, how their social interactions had been affected by the COVID-19 pandemic, and whether they were willing to conduct some of their voluntary activities using a remote-controlled robot. The results of this study show the potential of robots to replace some face-to-face interactions in organizing older adults' meaningful activities in times when their ability to have such face-to-face interactions is limited.

Martin et al. also investigate the role of robots to mitigate loneliness, offering a different perspective: people's views on the role of an artificial companion (AC) regarding deception with dementia and its role in reducing loneliness. The study raises some important concerns regarding the ethical issues of the current design solutions concerning artificial companions. The participants did not think that a companion robot would make them feel less lonely, and they felt that the deception of allowing people to believe that an artificial companion is human would make them feel uncomfortable. The participants challenged the role of a potential artificial companion in mitigating loneliness, the social desirability of such an innovative technology solution, and raised important moral concerns, regardless of their age and gender.

Racin et al. discuss the role of videoconferencing for older adults in nursing homes during the COVID-19 pandemic using the concept of “mediation”. Practices of interaction with families and friends using videoconference applications were revealed using interviews and observations undertaken among residents, their relatives, professionals, and the management teams, showing considerable inequalities in terms of skills, subjective feelings, and ownership of the videoconferencing tools. Although designed to increase the positive effect on older adults, social interactions, and wellbeing, the difficulties associated with the use of teleconferencing in nursing homes have emerged and have been deeply analyzed in this study, raising the question on dependency, protection, respect for people's autonomy, and failure to consider residents' feelings and disturbance in the situation.

Iancu and Iancu present insights into the perceptions of chatbots among adults in mid and later life based on a sample of 235 people ranging in age from 40 to 78 years. This study contributes new knowledge to an area that has to date primarily focused on younger people between 18 and 34 years of age because of the common belief of their experienced use of technologies. The findings from this study note the perceived ease of use and behavioral intention were important factors for using chat bots, especially if engagement was useful, in addition to positive feedback or opinions from other people. However, gender and age showed no effect in relation to behavioral intention.

Morrison et al. present a qualitative study and survey and an additional nine interviews to understand the issues and types of engagement experienced by older adults interacting with various digital practices, such as QR codes and paying for items using contactless methods and apps via smartphones. The findings, via a thematic analysis approach, highlighted two factors: (1) Intrinsic—digital literacy and (2) Extrinsic—technology glitch or breaking, which in turn lead to a reduced opportunity for social inclusion and feelings of embarrassment in the physical space. The digital divide continues to grow, and this study contributes knowledge to understanding how during the Fowe and Boot present a study focusing on technology use to facilitate remote monitoring and virtual care of patients and people, respectively, with a view to affording greater efficient and effective methods in our growing aging populations. A quantitative survey was deployed to 92 community-dwelling adults to explore their attitudes toward using wearable and mobile technologies associated to (1) predicting cognitive decline, (2) assisting with adherence to healthy activities, and (3) collecting self-report data to understand current and predict future health states. Overall, the findings ascertained that in theory, and from a hypothetical standpoint, digital solutions would be useful, and there was an interest to learn more and a willingness to adopt digital solutions for these purposes. However, the findings did show a neutral response regarding concerns associated to data privacy generated via the digital solutions. Further, these concerns showed a lesser interest and willingness to adopt digital technologies, while there were greater positive associations to acceptance and willingness to adopt digital technologies based on positive attitudes and proficiency with mobile devices. Respondents of the survey who self-reported to have poor health showed a negative attitude toward digital technologies, and this too highlights the barrier-targeting interventions to increase the adoption of digital technologies.

Wilson-Menzfeld et al. present findings from a study primarily focusing on the perceived facilitators and barriers toward a remote digital skills programme, and they ascertain whether this method of training could be utilized as an alternative to face-to-face methods. Their findings identified two key themes: (1) creating a unique learning environment and (2) encouraging further learning. While there were barriers to this mode of delivering the programme, they also identified positive factors, including the personalization and individuality of the programme delivery, which in turn empowered the participants within their own learning experiences, and skills were learnt that were relevant to the individual, resulting in the individuals continuing their digital learning.

Finkelstein et al. present findings from a study conducted in New York City and in conjunction with a multi-service organization to explore and understand the patterns and experiences of the adoption and use of digital technologies by older adults who had received devices, unlimited broadband, and technology training via the organization. Qualitative data were collected from 35 older adults who were in receipt of the digital devices, connectivity, and training, ranging between 55 and 90 years of age. Additional characteristics of the older adults highlighted were that they constituted a racially/ethnically diverse group (Black 29%, Latino 19%, White 43%) and all had low incomes. The findings from this study show that training programs designed with a one-size-fits-all approach do not necessarily work, and instead, training should be customized to reflect the skills that are needed instead of primarily basing it on age. The authors posit and recommend that service-led organizations should include and conduct technology assessments relating to access and use in other standard protocols.

Reuter et al. posit in their article the need to look toward the future in a post-pandemic society, and while digital inclusion is important and was highlighted during the pandemic, digital participation is also important to identify and augment opportunities for everyone in our communities and society. Moving forward, Reuter et al. argue the need to implement a macro-, meso-, and micro-level approach to enable and facilitate digital participation in later life, with a view to establishing a multifaceted and a multisectoral approach to partnerships associated to environmental factors. This approach has the potential to appropriately design and implement digital participation programmes, with additional evaluations to be considered concerning the needs and lived experience of older adults.

In this Research Topic, there is strong discourse surrounding digital skills, literacy, inclusion, attitudes toward and perceptions of digital technologies, and practices that are integral to current and future research investigations associated to digital technologies and practices. What is noticeable as we transition into a post-pandemic society, as we reflect upon the pandemic, is that many people in our communities and society in general were excluded from societal activities and access to vital information, as well as being able to access information via QR codes (e.g., restaurant menus) or government websites and other associated services pertinent to track and trace (Katz and Marshall, 2018; Beneito-Montagut et al., 2022; Rosales et al., 2023). Digital inclusion is imperative for everyone in society, especially as we take a transgenerational technology approach to interactions and perspectives (Rosenberg, 2019; Sourbati and Loos, 2019; Ivan and Cutler, 2021b; Fernández-Ardèvol and Grenier, 2022). However, many people have still been excluded because they do not have the confidence to use new technologies, or there are no digital programmes available for them to upskill their digital skills and literacy (Marston et al., 2020b; Sourbati and Behrendt, 2021).

The research presented contributes to the growing body of work in the fields of social sciences, gerontology, gerontechnology, health, and wellbeing, but greater efforts are needed. Digital skills and those people who are currently and likely to be excluded, or those who have greater challenges in our society because of their physical environment—such as living in rural or remote areas—financial implications (Dow-Fleisner et al., 2022), or access to knowledge (Marston and Van Hoof, 2019), continue to be ignored. This requires more effort from scholars. We must reduce echo chambers, reinvent the wheel, and instead include a broader group of people in research activities if there will be any attempt to actually understand why people from marginalized communities do not have the digital skills or ownership of digital devices, which, in turn, would assist them to conduct alternative modes of purchasing, or to extend their learning practices relating to digital technologies and practices. This has to be rectified.

Evidence-based research can play an integral role in this process, alongside co-designing and co-producing training manuals with people, young or old. One example we can draw on is from the “Adapt Tech, Accessible Technology” (ATAT, 2020–2022) project [2020–2022], conducted by researchers in the UK, engaging with various stakeholders and older adults online through a series of workshops, which resulted in several deliverables.

Much work has been undertaken regarding the digital divide, yet the narratives continue to be purported with little tangible solutions offered. Furthermore, the imbalance in accessing digital healthcare and health professionals as well as in complimenting social care needs must be reduced if digital technologies are meant to be the solution for managing greater remote (health) monitoring (Litchfield et al., 2021). Similarly, health professionals and people undertaking educational programmes who wish to work in the health and social care professions need to acknowledge and realize the need for and role of digital technologies in our daily lives. Therefore, such educational programmes should instill curricula associated to digital technologies and practices, enabling future practitioners the knowledge (at the minimum) and skills to feel confident to use within practice and the community (Dumitru et al., 2022).

The knowledge contributed to this Research Topic can and should benefit members of the wider scholarly communities, and we hope future investigations will take note of the respective findings published throughout the different articles published here. Additionally, we hope future readers of this Research Topic will realize that continuing to work within their own echo chambers is not conducive to the overall goals of reducing digital exclusion, enhancing digital inclusion, and actually planning for future aging populations, because Generation X and other younger cohorts have different needs and experiences of digital technologies and practices (Marston and del Carmen Miranda Duro, 2020; Loos and Ivan, 2023). Therefore, when younger generations reach later life, their expectations will differ to that of the current older population, and they will expect appropriate solutions.

## Author contributions

LI: Writing—review and editing. HM: Writing—review and editing. DR: Writing—original draft. BR: Writing—original draft.
